# Validation of the western ontario rotator cuff index in patients with arthroscopic rotator cuff repair: A study protocol

**DOI:** 10.1186/1471-2474-12-64

**Published:** 2011-03-31

**Authors:** Ronald N Wessel, Tjoan E Lim, Henk van Mameren, Rob A de Bie

**Affiliations:** 1 Department of Orthopedic Surgery, St. Antonius Ziekenhuis, PO Box 2500, 3430 EM Nieuwegein, The Netherlands; 2 Department of Orthopedic Surgery, Máxima Medical Center, PO Box 7777, 5500 MB Veldhoven, The Netherlands; 3 Department of Epidemiology, Caphri research school, Maastricht University, PO BOX 616, 6200 MD, Maastricht, The Netherlands

## Abstract

**Background:**

Arthroscopic rotator cuff repair is described as being a successful procedure. These results are often derived from clinical general shoulder examinations, which are then classified as 'excellent', 'good', 'fair' or 'poor'. However, the cut-off points for these classifications vary and sometimes modified scores are used.

Arthroscopic rotator cuff repair is performed to improve quality of life. Therefore, disease specific health-related quality of life patient-administered questionnaires are needed. The WORC is a quality of life questionnaire designed for patients with disorders of the rotator cuff. The score is validated for rotator cuff disease, but not for rotator cuff repair specifically.

The aim of this study is to investigate reliability, validity and responsiveness of WORC in patients undergoing arthroscopic rotator cuff repair.

**Methods/Design:**

An approved translation of the WORC into Dutch is used. In this prospective study three groups of patients are used: 1. Arthroscopic rotator cuff repair; 2. Disorders of the rotator cuff without rupture; 3. Shoulder instability.

The WORC, SF-36 and the Constant Score are obtained twice before therapy is started to measure reliability and validity. Responsiveness is tested by obtaining the same tests after therapy.

## Background

Arthroscopic rotator cuff repair is performed with increasing frequency. In more than 90% of the patients results are reported to be good to excellent[1-9]. However, the question remains what good to excellent really means. Especially, the University of California Los Angeles (UCLA) Shoulder Rating Scale and Constant Score are used frequently [1-9].

Authors use these instruments to classify results as 'excellent', 'good', 'fair' or 'poor'. Unfortunately the cut-off points vary and some authors use modified scores, making it difficult to compare results [1,4,5,8,10-12] as illustrated in Table 1.

**Table 1:** Publications considering rotator cuff repair using various versions of UCLA Shoulder Rating Scale and Constant Score and different cut-off points to translate the score into a qualification.

Article	Study population	Score	Translation to quality	Result
Tauro 1998[4]	53 arthroscopic RC repair	Modified UCLA	Good: 36-40 Excellent: 41-45	92% good/excellent

Gartsman 1998[10]	73 arthroscopic RC repair	UCLA Constant Patient satisfaction as item of the UCLA ASES SF-36	UCLA: Poor: 0-20 Fair: 21-27 Good: 28-33 Excellent: 34-35	UCLA: 84% good/excellent Satisfaction: 90% good/excellent

Burkhart 2001[8]	59 arthroscopic RC repair	Modified UCLA	Poor: < 29 Good: 29-33 Excellent: 34-35	95% good/excellent

Wolf 2004[5]	95 arthroscopic RC repair	Modified UCLA	Poor: 0-20 Fair: 21-27 Good: 28-33 Excellent: 34-35	94% good/excellent

Iannotti 1996[11]	46 open RC repair	Adjusted Constant Score pain and function questionnaire	Poor: < 70 Fair: 70-79 Good: 80-89 Excellent: 90-100	88% good/excellent

Lam 2004[12]	69 open repair massive RC tears	Constant Score Oxford Shoulder Questionnaire	Poor: < = 50 Fair: 51-65 Good: 66-80 Excellent: > = 81	Constant: 44% good/excellent

Boileau 2005[1]	65 arthroscopic RC repair	Adjusted Constant Score UCLA MRI	No translation described	Constant: 92% good/excellent

The UCLA Shoulder Rating Scale is a combination of physical examination and subjective evaluation of complaints of patients by the attending physician. No publications are available with respect to the development and validation of this instrument. Therefore it is unknown why the developers of this instrument assigned the various weights to the 5 domains. While not necessarily incorrect, it is thus far unsupported[13]. The Constant Score also consists of a combination of physical examination and subjective evaluation filled out by the attending physician. The publication by Constant et al[14]. does not describe the methodology according to which the instrument was developed and more specifically, the rationale for item selection and relative weighing of the items[13].

Both scores are filled out by the attending physician. Kirkley et al. stated that it had been shown that physicians tend to evaluate their patients functioning better than the patients perceive it themselves, making it important for measurement tools to be self-administered[15].

Validated outcome measures need to be used in randomized controlled trials of interventions for rotator cuff tears. In such trials, disease specific quality of life questionnaires could be a core outcome domain [16] in addition to pain, shoulder specific function, and work ability. Most therapies in orthopaedic medicine are designed to improve quality of life rather than to prolong a patient's life. Therefore, it is important to assess quality of life when such therapies are evaluated. Arthroscopic rotator cuff repair is also performed to improve quality of life, which depends on shoulder functioning relevant for day-to-day activities. Hence, health-related quality of life patient-administered questionnaires (HRQL-questionnaires) are needed[15]. The Western Ontario Rotator Cuff Index (WORC) is a disease-specific quality of life questionnaire (QoL-questionnaire) designed for patients with disorders of the rotator cuff (DRC), which was published in 2003 [15] with a clear description on how the items were chosen, formulated and weighted. For further details of WORC we refer to the appendix section.

The WORC is used in research [17] and has already been translated into German [18], Iranian [19], Norwegian [20], Portuguese [21] and Turkish [22]. A Dutch translation of the WORC will be used in our clinic. This translation was performed by linguistic validation, which is not a literal translation but a stepwise process to produce a conceptually equivalent of the original questionnaire[23]. The process was controlled with final approval by one of the authors of WORC[15]. In this study the Dutch version of the WORC is validated.

As far as we know the WORC has not been tested on a group of patients with arthroscopic rotator cuff repair. In the WORC article by Kirkley et al[15]. validity as an evaluative tool was tested in a group of 50 patients with disorders of the rotator cuff (DRC) who reported that their symptoms had changed. Forty-six were treated with injection therapy and only four underwent subacromial decompression[15]. In order to use the test in those who underwent arthroscopic rotator cuff repair, the WORC should be tested in a group of patients treated with this type of repair.

The aim of this study is to investigate reliability, validity and responsiveness of WORC in patients undergoing arthroscopic rotator cuff repair. Because there is no gold standard we are aiming to assess construct validity, i.e. the extent to which WORC scores relate to SF-36 and Constant Scores. More experiences with the disease specificity of the use of the WORC are needed, since this instrument is designed for use in patients with DRC only.

Therefore, three groups of patients are chosen in which the WORC will be tested. These groups contain patients with respectively:

1. Arthroscopic rotator cuff repair;

2. DRC without rupture;

3. Shoulder instability.

We hypothesize that the Dutch translation of WORC:

• has a high reliability (ICC > 0,9) in all 3 groups;

• has a high validity compared to SF-36 and Constant Scores in groups 1 and 2. We expect correlations on the SF-36 and Constant Score to lie around 0.6 and an inverse correlation between Constant Score and SF-36 versus WORC around -0.5[22]; The relation is expected to be negative due to the construction of WORC, in which a high score corresponds to a disabled shoulder, while a high Constant Score and SF-36 match with little disability.

• has a high responsiveness in groups 1 and 2;

• will be more specific to determine the extent of complaints and change in time supposed to be caused by therapy in patients in the groups 1 and 2 when compared to patients in group 3.

It is the experience of orthopaedic surgeons, also mentioned in textbooks[24,25] that patients with a rotator cuff tear (group 1) suffer pain and weakness when they use the ruptured part of the rotator cuff. In patients with DRC (group 2) patients show tendonitis resembling, more continuous, pain. For instance in this group, inability to raise the arm is rather caused by pain than by lack of strength. Patients with instability (group 3) in general complain about dislocations, subluxations or a sense of instability[26,27]. For this group of patients it is easier to avoid provocative movements compared to patients with rotator cuff disease.

The WORC consists of 5 domains: (1) physical symptoms, (2) sports and recreation, (3) work, (4) social function, and (5) emotions. We expect that the scores for each domain will not differ significantly from each other in group 1 and 2, because Kirkley stated that the frequency importance products of the 21 items have a narrow range[15]. On the contrary, based on our experience as orthopaedic surgeons, we assume that especially the domains physical symptoms (1) and social function (4) in group 3 will be rated better, because in the first domain many items involve pain and in the 4^th ^domain the provocative actions would be avoided easier by patients with instability. Resulting from these expectations, we assume that the responsiveness in group 3 will be low for domain 1 and 4.

Unique in our study design is that the patient selection and assignment into subgroups is performed in a continuing clinical process, including both interventions at the outpatients' department and surgical interventions. In this dynamic clinical setting patients can be transferred between the subgroups and inclusion can be performed continuously.

## Methods/Design

### Patient assignment

The WORC, as a disease-specific evaluation instrument, was developed for patients with a DRC[15]. In order to investigate reliability, validity and responsiveness of the WORC in patients with arthroscopic rotator cuff repair, data from patients undergoing an arthroscopic rotator cuff repair are analysed (group 1). To investigate disease-specificity of the WORC when compared to a different DRC, a second group 'non-Ruptured DRC' is added (group 2). This group includes the rest of rotator cuff pathology like rotator cuff tendonitis and tendinosis[15]. Bursitis and calcifying tendonitis are also included in non-Ruptured DRC. A third group consisting of patients with 'Shoulder Instability' is included in order to investigate the disease-specificity of WORC when compared to a different shoulder disease (group 3). Each group will consist of 30 patients in which we assume that at least one group will differ 25% on WORC score from the other groups, which yields an 80% power with a one sided α of 5%. This would also provide ample power for test-retest reliability with correlation coefficient of at least 0.95[28].

#### Signs and symptoms

Group-1: Patients undergoing arthroscopic rotator cuff repair. These patients have a clinical suspicion of a symptomatic rotator cuff tear and a partial or full thickness lesion seen on MRI or Ultrasound and confirmed during the arthroscopic repair procedure. A clinical suspicion will be considered present in case of two or more of the following signs: Impingement (Neer's impingement sign or Hawkins-Kennedy impingement sign), Painful arc sign, positive Jobe test (supraspinatus), positive infraspinatus test (resistance test with external rotation at the side and in 90 degrees of abduction), positive lift-off/belly press test (resistance test of subscapularis) [29], positive Drop-arm test [30], positive Neer impingement test [29] (subacromial injection with lidocaine).

Group-2: Patients diagnosed as having non-Ruptured DRC. They have a clinical suspicion of DRC and no rupture of the rotator cuff on MRI or Ultrasound. A clinical suspicion will be considered present in case of two or more of the following signs: Impingement (Neer's impingement sign or Hawkins-Kennedy impingement sign), Painful arc sign, positive Jobe test (supraspinatus), positive infraspinatus test (resistance test with external rotation at the side and in 90 degrees of abduction), positive lift-off/belly press test (resistance test of subscapularis) [29] and positive Neer impingement test (subacromial injection with lidocaine). Patients are also assigned to Group-2 if the symptoms are accompanied by calcification of the tendon close to the greater tuberosity seen on X-ray. In that case further imaging like MRI or Ultrasound is not performed necessarily.

Group-3: Patients diagnosed with shoulder instability. They fulfil at least three of the following criteria: history of gleno-humeral dislocation, history of sense of instability, positive apprehension test, positive relocation test, glenohumeral translation, positive sulcus sign [29], positive jerktest [31], positive hyperabduction test [32], labral lesion on MRI, capsular lesion or laxity on MRI.

For a clear description of the clinical tests we refer to the appendix section.

#### Patient routine

Prior to the first visit to the orthopaedic shoulder outpatient clinic, consecutively, all new patients above age 18 are informed about the study by telephone. Patients who have difficulty understanding the explanation, because of language deficiency are excluded. The patient routine contains three measurement moments T0, T1 and T2.

During the first visit (T0), all new patients are asked whether they want to participate in the research project. If the answer is positive, the patients sign an informed consent form. Then a thorough history is taken and the patients will undergo a physical examination including the above mentioned clinical tests by the second author (TEL). Subsequently, scores on the WORC, SF-36 and the Constant Score are obtained, and patients are asked to rate their shoulder hindrance on an 11-point scale from 0 (no hindrance) to 10 points (extreme hindrance), all by the second author (TEL). (See the appendix section for details on obtaining the Constant Score.) In case of suspicion of instability or a cuff tear MRI is performed. If MRI is contraindicated and a cuff tear is suspected ultrasound is performed. Patients do not receive any therapy at T0.

The next visit is scheduled 2-3 weeks after T0. This visit is defined as T1. At T1 the WORC, SF-36 and Constant Score are scored again. In addition, the patients are asked to rate their shoulder hindrance on the 11-point scale to check whether their symptoms have changed since their last visit.

At T1 either conservative treatment is started or the indication for surgery is made.

In Group 1 an arthroscopic rotator cuff repair is performed. In case of an irreparable cuff tear due to size, atrophy or fatty degeneration the patient is excluded from the study.

In Group 2 conservative treatment is started by injection, physiotherapy and activity adjustment. Surgery is performed when conservative treatment failed. Arthroscopic subacromial decompression is in those cases performed and in patients with calcific tendonitis the calcium deposit is removed. If during arthroscopy an asymptomatic cuff tear is found it is repaired, but these patients are then excluded from the study.

In Group 3 patients receive conservative treatment or surgery when indicated. A labral or capsular repair is performed as needed.

Patients eligible for assignment to more than one group (e.g., those with both instability and impingement) are excluded from our study, because specificity of the WORC between the groups cannot be measured.

Patients with additional shoulder pathology like adhesive capsulitis, AC-pathology and osteoarthritis are excluded and patients with previous surgery are excluded as well.

At T2, either six weeks after starting conservative therapy or three months after operative therapy, the WORC, SF-36 and Constant Score are obtained again.

Patient characteristics like age, sex, hand dominance and Workers' Compensation claim are recorded and taken into account during data analyses, because we are comparing groups. If power permits we will control these group-characteristics.

Data will be analyzed with SPSS 15. Retrieved data from T0 and T1 will be checked for completeness and entered into Excel.

### Reliability

Reliability of the WORC is tested by comparing the results at T0 with T1. T1 is planned 2-3 weeks after T0, because we expect that the symptoms do not change between these two moments and the time span is large enough to forget initial responses to the questions. In order to signal change in severity of the symptoms, at both moments patients are asked to rate their shoulder hindrance. To determine test-retest reliability, intraclass correlation coefficients (ICC) are used.

### Validity

Criterion validity is measured by comparing WORC with a general quality of life questionnaire (SF-36) and a commonly used clinical shoulder score (Constant Score) both at T0 and T1. Bland Altman plots will be used to estimate 95% boundaries of concurrence.

### Responsiveness

Responsiveness, or the ability of an instrument to detect clinically important change, will be determined by calculating the standardized response means and effect sizes on T0 and T2 scores on WORC, SF-36 and Constant scores for the subgroups. The magnitude of the standardized response mean (mean change in score from T0 to follow-up (T2)/standard deviation of change in score) and the effect sizes (mean change in score from T0 to follow-up (T2)/standard deviation of T0 score) will be interpreted using the Cohen standard of greater than 0.20 for small effects, greater than 0.50 for moderate effects, and greater than 0.80 for large effects.

Finally, a comparison of WORC scores between the three distinct groups is made, to see whether WORC scores can differentiate between them. WORC scores will be compared with SF-36 to investigate whether the noted differences correlate with notable clinical differences and can be expressed as MCID's (minimal clinical important differences)[33].

### Ethical Approval

Ethical approval from The Institutional Review Board/Independent Ethics Committee (IRB/IEC) of Máxima Medical Centre has been obtained.

## Discussion

Evidence on efficiency and safety of arthroscopic rotator cuff repair is not available from randomized controlled trials. Based on case series this treatment is often referred to as being a successful procedure with high rates of excellent to good results[1-9]. These conclusions are often derived from clinical scores like the UCLA Shoulder Rating Scale and the Constant Score. A disadvantage of these scores is that they are often filled out by the attending physician, making the results vulnerable for bias. Patient administered questionnaires will bypass this problem.

We choose the WORC because of the following reasoning:

Many questionnaires concerning shoulder pathology have been used, e.g. Disabilities of the Arm, Shoulder and Hand (DASH),[34] Simple Shoulder Test (SST),[35] Shoulder pain and disability index (SPADI),[36] The Shoulder Rating Questionnaire by L'Insalata et al.,[37] "Oxford" questionnaire on the perceptions of patients about shoulder surgery,[38] Rowe Score,[39] The American Shoulder and Elbow Surgeons Score (ASES)[40], The Rotator Cuff Quality-of-Life Measure (RC-QOL),[41] and the Western Ontario Shoulder Tools (WORC, WOOS, WOSI)[13]. Disease-specific instruments are encouraged to be used, when available, to improve sensitivity to change[16].

Only two of the above mentioned scores are disease-specific QoL-questionnaires concerning rotator cuff pathology, The Rotator Cuff Quality-of-Life Measure (RC-QOL) [41] and The Western Ontario Rotator Cuff Index (WORC)[15].

The WORC is primarily composed by and for people with Rotator Cuff Disease and the way it is developed including item selection, formulation and weighting is adequately described[13]. We prefer the WORC to RC-QOL, because it has already been translated into a number of languages[18-22] and has been used in various studies [42-53], which makes it interesting for international comparison.

In the presenting article of the WORC in 2003, the instrument is validated by correlating the questionnaire to various shoulder measures and the SF-36 in a group of 50 patients. However, none of them underwent arthroscopic rotator cuff repair[15]. Nonetheless, the WORC is used for measuring the result rotator cuff repair[42,43,45,48,49].

By now, arthroscopic rotator cuff repair is a commonly accepted treatment. We also want to use the WORC to measure the results of our arthroscopic rotator cuff repairs. In patients with DRC the WORC is an appropriate measurement tool[15]. We will investigate whether this also accounts in patients with arthroscopic rotator cuff repair. To do so we investigate a group of patients with arthroscopic rotator cuff repair. A second group is used existing of patients with DRC analogical to the group used by the developers of the WORC. And in order to investigate specificity within the shoulder pathologies it is tested on a group of patients with a different type of shoulder complain, instability.

Reliability is tested by comparing T1 and T0 in the three groups. Concerning validity we are aiming to assess construct validity, i.e. the extent to which WORC Scores relate to SF-36 and Constant Scores. Construct validity can be assessed by comparing the convergent/discriminant validity across instruments and patient groups ('known-groups validation', hence the three patient groups). It should be noted that the study will not truly be able to assess disease specificity of the WORC because the three patient groups differ not only with respect to diagnosis, but also with respect to treatment modality. The Constant Score was elected to correlate with, because it is a clinical score which is commonly used and widely accepted in Europe[13]. Responsiveness is tested by comparing T2 and T0.

The name of the WORC implies specificity to rotator cuff disease, although it has been developed to assess quality of life regarding DRC, and not as a discriminative tool[15]. Therefore, the results of the WORC in group 1 and 2 are compared with a third group consisting of patients with instability to investigate specificity.

The method of patient assignment could be a subject of criticism. Patients were assigned to a certain group based on a combination of clinical symptoms and findings on MRI, Ultrasound or X-ray, because the clinical tests for DRC and shoulder instability lack high accuracy[30,54-58]. However, this was then confirmed during arthroscopy.

The time interval between therapy and T2 depends on the type of therapy, conservatively or surgically. This can give the impression of surgery being too favourable. The goal in this study is among others to measure the responsiveness of the WORC, and not to compare different kinds of therapy. The result of conservative treatment can be measured after 6 weeks. In case of success, treatment will be finished. We believe that extra visits after finishing treatment are of increasing load for patients leading to withdrawal and accompanying bias.

The dynamic design of this study concerning continuing inclusion and possible transferring of the patients in between subgroups makes it possible to perform a validation study in a continuing clinical setting avoiding high costs. The disadvantage of transferring patients between subgroups during the study is that it makes the study more vulnerable for bias. On the other hand, in this prospective design, rules concerning changing and exclusion have been clearly described.

This is the first prospective study to gain further insight into the validity of the WORC in patients treated arthroscopically for rotator cuff lesions. In this study the Dutch version of the WORC is validated, which will provide the use of an international comparable disease-specific QoL questionnaire concerning rotator cuff pathology. The dynamic design of this validation study with subgroups in a continuing clinical process is unique and can be used for validation of many system-specific and disease-specific outcome instruments in many medical disciplines.

## Competing interests

The authors declare that they have no competing interests.

## Authors' contributions

The authors' responsibilities were as follows. RNW: study design, data collection, data analysis, and writing of the manuscript; TEL: study design, data collection, and review of the manuscript; HvM: study design, review of the manuscript; and RAB: study design, data analysis, and review of the manuscript. All authors read and approved the final manuscript.

## Appendix

### Description of WORC and item selection

The Western Ontario Rotator Cuff Index (WORC) [15] is designed for patients with disorders of the rotator cuff[15]. It is an HRQL-questionnaire that has 21 items representing 5 domains, each with a visual analogue scale-type response option. The 5 domains are (1) physical symptoms, (2) sports and recreation, (3) work, (4) social function, and (5) emotions. WORC items are scored on a 100-point scale (ranging from 0-100). The most symptomatic score is 2100, and the best, or asymptomatic, score is 0. To present this in a more clinically meaningful format, the score can be reported as a percentage of normal by subtracting the total from 2100, dividing by 2100, and multiplying by 100. Total final WORC scores can, therefore, vary from 0%, the lowest functional status level, to 100%, the highest functional status level [59].

Items for the WORC were derived from published health status scales, functional measures of the shoulder, discussions with healthcare professionals, and interviews with 30 patients from a registry of 150 with rotator cuff pathology. Both professionals and patients were asked to identify ways in which the shoulder condition affected quality of life in general and the 5 domains in particular. The 30 patients interviewed included males and females, aged 30-76, with different degrees of rotator cuff pathology from tendinitis to massive tears.

An original list of 321 items was reduced to 76 by the investigators eliminating duplicated, incomprehensible or ambiguous items. A random selection of 100 patients from the same registry were then asked to indicate whether they experienced each of the items, and to rate the importance of the symptom/disability to their overall shoulder functioning. A frequency importance product was calculated for each item and the 50 items with the highest values were correlated with each other. For every pair of items with coefficients greater than 0.6, one of the items was eliminated, resulting in the final 21 questions. It is not clear whether this criterion applied to items across domains because the only example provided included 3 items from the same domain[60].

### Constant Score

The Constant score was devised by Christopher Constant with assistance of Alan Murley. The score was first presented in a university thesis in 1986 and the methodology published in 1987[61].

The Constant score purely assesses how well a shoulder functions by choosing a number of functional parameters: pain, activities of daily living, elevation, range of movement and strength. Constant states that there is a wide discrepancy between normal values from different centers, because there is a great deal of uncertainty as to the methodology. Therefore, we will administer the score as advised by Constant et al[61]. Strength will be measured by an electronic device (Kinedyne myometer). The maximum of 3 repetitions, each separated by 1 minute, will be recorded.

### Clinical tests

#### Impingement

Impingement is considered positive if the Neer's impingement sign or Hawkins-Kennedy impingement sign is present. Neer's impingement sign is positive if a forcible elevation of the arm with the scapula stabilized causes the pain[29].

The Hawkins-Kennedy impingement sign involves forward flexing the humerus to 90° and forcibly internally rotating the shoulder. This manoeuvre drives the greater tuberosity under the coracoacromial ligament similarly reproducing the impingement pain[29].

The Neer impingement test documents the patient's response to an injection of lidocaine into the subacromial bursa. An attempt to elicit the impingement sign is repeated. A significant reduction or abolition of the patient's pain constitutes a positive result of the impingement test[29].

#### Painful arc

The patient is asked to actively abduct the arm. If the patient cannot do this actively, attempt to do this passively, remembering to rotate the arm externally while doing so. Ask the patient to lower the arm to the side [62]. The test is considered to be positive if the patient experiences pain or painful catching between 60° and 120° of elevation[56].

#### Jobe

The supraspinatus tendon is tested by the Jobe test which is a resistance test with the arm abducted to 90° in the plane of the scapula and the forearm maximally pronated [29]. The resistance test is considered positive when a clear weakness is demonstrated during muscle strength testing against resistance[56].

#### Infraspinatus tendon

The infraspinatus tendon is tested by resisted external rotation at the side and in 90 degrees of abduction[29]. The resistance test is considered positive when a clear weakness is demonstrated during muscle strength testing against resistance.

#### Lift-off test

The subscapular tendon is tested by the lift-off test described by Gerber and Krushel. The dorsum of the ipsilateral hand is placed on the sacrum and the patient must "lift off" the hand from the back while the examiner maintains the elbow in the coronal plane. The test is abnormal if the patient is unable to lift the hand off the back. This test can only be interpreted accurately if the patient has a full range of *passive *internal rotation (the hand can be passively lifted off the back) and if *active *internal rotation is not limited by pain[29,63].

If the test cannot be interpreted accurately, the belly press test is used.

#### Belly Press Test

In this test the patient presses the abdomen with the flat of the hand and attempts to keep the arm in maximal internal rotation. If active internal rotation is strong, the elbow does not drop backward, meaning that it remains in front of the trunk. If the strength of subscapularis is impaired, maximum internal rotation cannot be maintained, the patient feels weakness, and the elbow drops back behind the trunk. The patient exerts pressure on the abdomen by extending the shoulder rather than by internally rotating it[29].

#### Drop Arm test

The patient is asked to elevate the arm fully. If it is not possible to do this actively, passive motion will be attempted. Then the patient is asked to slowly reverse the motion in the same arc. If the arm drops suddenly or the patient has severe pain, the test is considered to be positive[56,62].

#### Apprehension test

This test can be performed when the patient is either in a standing or a supine position. As the shoulder is moved passively into maximum external rotation in abduction and forward pressure is applied to the posterior aspect of the humeral head, the patient suddenly becomes apprehensive and complains of pain in the shoulder[29].

#### Relocation test

Repeat the apprehension test with the patient in the recumbent position; abduct and externally rotate the shoulder. When pain or apprehension first appears, press down on the upper arm. This will stabilize the head of the humerus in the glenoid at the time when subluxation is imminent, and should relieve any pain or apprehension. This, and the return of pain and apprehension on release of the downward pressure, is confirmatory of anterior instability[62].

#### Glenohumeral translation

This test is considered positive if the patient's symptom complex can be reproduced during translation manoeuvres[29].

#### Hyperabduction test

In order to measure the range of passive abduction (RPA), the physician stands behind the patient with his forearm pushed down firmly on the shoulder girdle in its lowest position, while lifting the relaxed upper limb in abduction with his other hand. During the test, the elbow is flexed at 90° and the forearm is horizontal. An RPA of more than 105° is associated with lengthening and laxity of the inferior glenohumeral ligament.

## Pre-publication history

The pre-publication history for this paper can be accessed here:

http://www.biomedcentral.com/1471-2474/12/64/prepub
